# Development of Combination Methods for Detecting Malignant Uptakes Based on Physiological Uptake Detection Using Object Detection With PET-CT MIP Images

**DOI:** 10.3389/fmed.2020.616746

**Published:** 2020-12-23

**Authors:** Masashi Kawakami, Kenji Hirata, Sho Furuya, Kentaro Kobayashi, Hiroyuki Sugimori, Keiichi Magota, Chietsugu Katoh

**Affiliations:** ^1^Graduate School of Biomedical Science and Engineering, Hokkaido University, Sapporo, Japan; ^2^Department of Diagnostic Imaging, Graduate School of Medicine, Hokkaido University, Sapporo, Japan; ^3^Faculty of Health Sciences, Hokkaido University, Sapporo, Japan; ^4^Division of Medical Imaging and Technology, Hokkaido University Hospital, Sapporo, Japan

**Keywords:** object detection, deep learning, positron emission tomography, YOLOv2, computer vision

## Abstract

Deep learning technology is now used for medical imaging. YOLOv2 is an object detection model using deep learning. Here, we applied YOLOv2 to FDG-PET images to detect the physiological uptake on the images. We also investigated the detection precision of abnormal uptake by a combined technique with YOLOv2. Using 3,500 maximum intensity projection (MIP) images of 500 cases of whole-body FDG-PET examinations, we manually drew rectangular regions of interest with the size of each physiological uptake to create a dataset. Using YOLOv2, we performed image training as transfer learning by initial weight. We evaluated YOLOv2's physiological uptake detection by determining the intersection over union (IoU), average precision (AP), mean average precision (mAP), and frames per second (FPS). We also developed a combination method for detecting abnormal uptake by subtracting the YOLOv2-detected physiological uptake. We calculated the coverage rate, false-positive rate, and false-negative rate by comparing the combination method-generated color map with the abnormal findings identified by experienced radiologists. The APs for physiological uptakes were: brain, 0.993; liver, 0.913; and bladder, 0.879. The mAP was 0.831 for all classes with the IoU threshold value 0.5. Each subset's average FPS was 31.60 ± 4.66. The combination method's coverage rate, false-positive rate, and false-negative rate for detecting abnormal uptake were 0.9205 ± 0.0312, 0.3704 ± 0.0213, and 0.1000 ± 0.0774, respectively. The physiological uptake of FDG-PET on MIP images was quickly and precisely detected using YOLOv2. The combination method, which can be utilized the characteristics of the detector by YOLOv2, detected the radiologist-identified abnormalities with a high coverage rate. The detectability and fast response would thus be useful as a diagnostic tool.

## Introduction

Deep learning technology has developed rapidly and is now used in real-world settings such as automated driving, games, image processing, and voice recognition (1–4). Deep learning has also been applied to the field of medical imaging; e.g., in the classification of computed tomography (CT) images in different slice positions (5) and its training algorithm (6), research concerning the diagnosis and processing of pulmonary nodules by deep learning for feature extraction, detection, false-positive reduction, and benign malignant classification (7), and a study using deep learning to improve the performance of the automatic detection of lesions on mammograms (8). In such applications, knowledge of anatomy is required to process and diagnose medical images, and an inexperienced person may not be able to process and diagnose the images appropriately. However, if automatic object detection using deep learning (9–14) can be used for medical imaging, it could be possible to perform highly reproducible processing without the requirement of the knowledge and experience of physicians and radiologists.

Fluorodeoxyglucose-positron emission tomography (FDG-PET) (15) is an imaging method in which FDG labeled with fluorine-18 (F-18) is injected into the body, and two 511 keV annihilation photons which are produced by the positron decay of F-18 are simultaneously injected into the opposing detectors and reconstructed. FDG is an analog of glucose and accumulates in tumors with increased glucose metabolism as well as in organs *in vivo*, such as the brain, where glucose consumption as energy is high. It is therefore necessary to determine whether each site of high FDG uptake is a physiological uptake or an abnormal uptake. It has been demonstrated that a convolutional neural network (CNN) was useful for classifying FDG-PET images into normal, abnormal, and equivocal uptakes (16). In the present study, we investigated the precision of an object detection model, You Only Look Once version 2 (YOLOv2) (17), which uses deep learning to automatically detect the physiological uptakes on maximum intensity projection (MIP) images of FDG-PET in a rectangular region. We also developed a combination method to generate images in which abnormal uptakes were enhanced by subtracting only the detected physiological uptakes from the original MIP images. For an evaluation of the potential clinical uses of this combination method, we calculated the coverage rate by comparing the generated images to the abnormal uptakes that were identified by previous imaging findings.

## Materials and Methods

### Subject and PET-CT Scans

The study included a total of 500 patients (287 males and 213 females, age 61.3 ± 17.0 years [mean ± SD]) who underwent a whole-body FDG-PET examination for the screening of malignant tumors between January and May 2016 at our institute. All MIP images were acquired using either a GEMINI TF64 PET-CT scanner (Philips Healthcare, Cleveland, OH, USA), or a Biograph64 PET-CT scanner (Siemens Healthcare, Erlangen, Germany). This study was approved by our institute's Ethics Committee [#017-0365].

### Automatic Detection

#### The Creation of the Datasets

A total of 3,500 MIP images of the 500 patients (seven images per patient at every 10° to ±30° from the front) were generated in the workstation equipped with the PET-CT scanners. We defined five classes of physiological uptake to be automatically detected: brain, heart, liver, kidney, and bladder. All image data were converted from Digital Imaging and Communications in Medicine (DICOM) files to Joint Photographic Experts Group (JPEG) files for further use.

**Figure 1:**
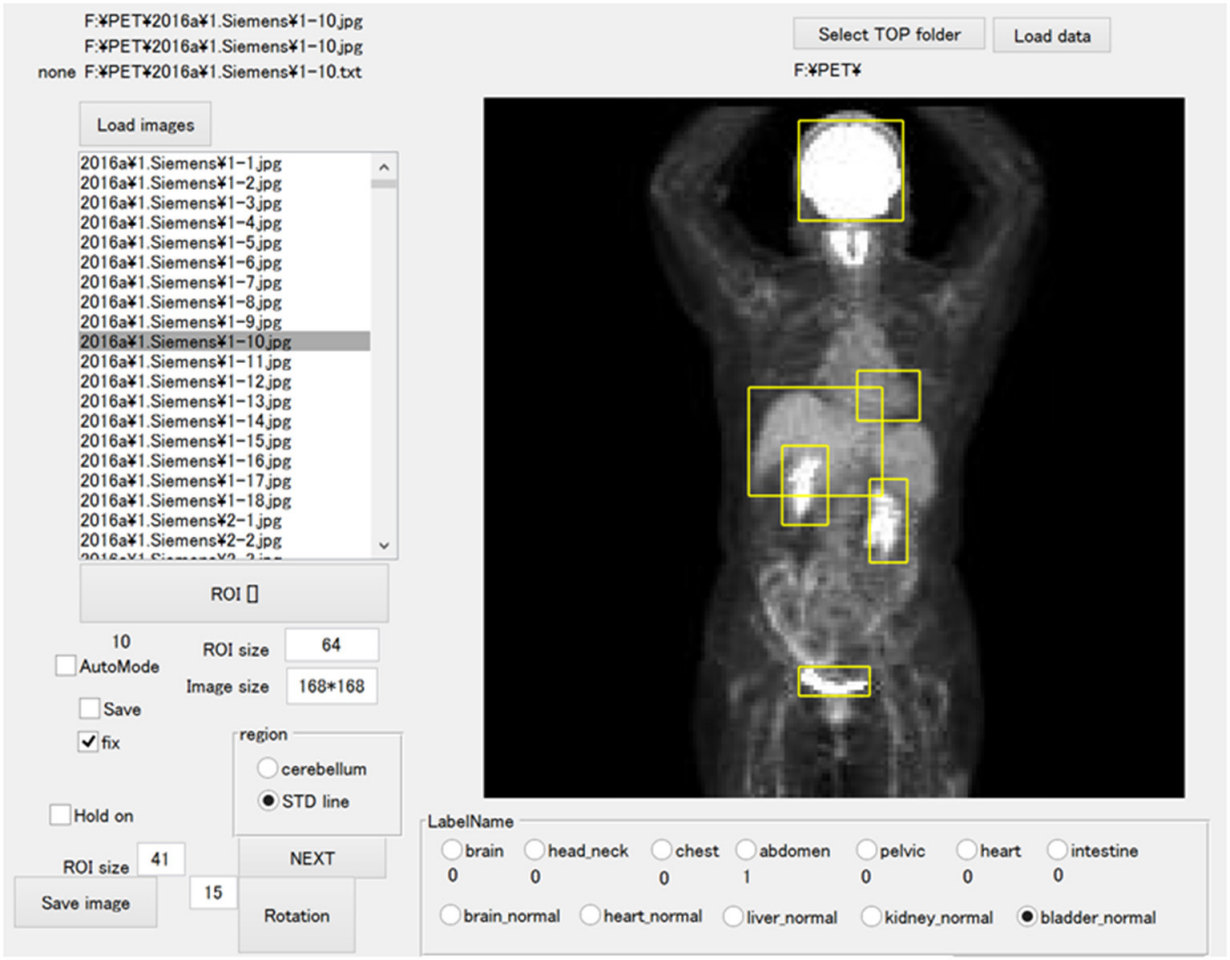
The software for training outlined ROIs over the physiological uptakes. The yellow bounding boxes enclose physiological uptakes.

**Figure 2:**
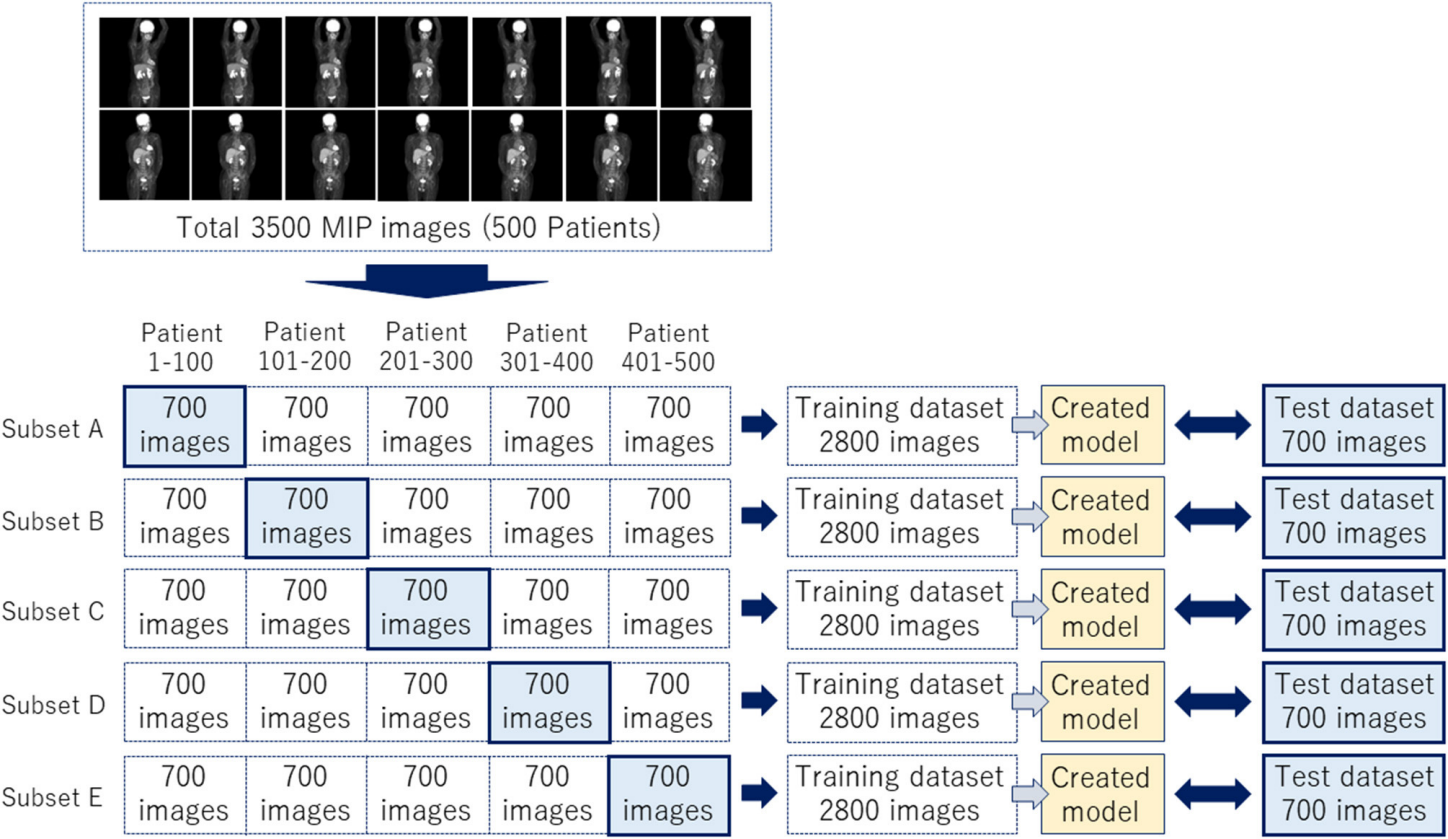
A total of 3,500 images were divided into five subsets to complete a nested cross-validation.

The JPEG files were loaded into the in-house MATLAB software program (MATLAB2019b, The MathWorks, Natick, MA, USA), and this program was used to draw rectangular regions of interest (ROIs) to enclose each physiological uptake (Figure 1). The ROI data were outputted as a text file, which included the object name, the coordinates, and the size of each ROI. We divided the supervised data into five subsets for nested cross-validation (18). Each 100 patients contributed 700 images; we used 2,800 images from 400 patients for training, and the remaining 700 images for testing (Figure 2). Each subset was an independent combination of 400 patients for training and 100 patients for test images, to prevent the mixing of patient images between the training and testing images within the subsets. To effectively learn for the training dataset, we performed data augmentation (19, 20) using image rotation from −15° to 15° in 3° steps and a zoom rate from 0.9 to 1.1 in 0.1 steps.

#### Training Images for Model Creation

We developed a software program for object detection with a deep learning technique via the in-house MATLAB software; we used a deep learning-optimized machine with an Nvidia Quadro P5000 graphics card (Nvidia Corp., Santa Clara, CA), which provides 8.9 Tera floating-point single-precision operations per sec, 288 GB/sec memory bandwidth, and 16 GB memory per board. We performed the image training as transfer learning by initial weight using YOLOv2, with the MATLAB deep learning Toolbox and Computer Vision System Toolbox. The training model hyperparameters were as follows: maximum training epochs, 10; initial learning rate, 0.00001; mini-batch size, 96. We used stochastic gradient descent with momentum for optimization with an initial learning rate. We set the momentum and L2 regulation to 0.9 and 0.0001, respectively. We performed image training five times based on the training subsets shown in Figure 2.

#### Evaluation of the Created Models

We incorporated the predicted bounding boxes into the MATLAB software in order to reveal the region of each physiological uptake as a bounding box. We also evaluated each physiological uptake by determining the average precision (AP), the mean average precision (mAP) (21), and the frames per second (FPS) for an estimation of the efficiency of the created model. The AP and mAP values were calculated by each intersection over union (IoU). We examined the bounding boxes based on the supervised ROI according to the “evaluateDetectionPrecision” and “evaluateDetectionMissRate” functions in the MATLAB Computer Vision Toolbox.

### Combination Method

#### The Creation of Color Maps

**Figure 3:**
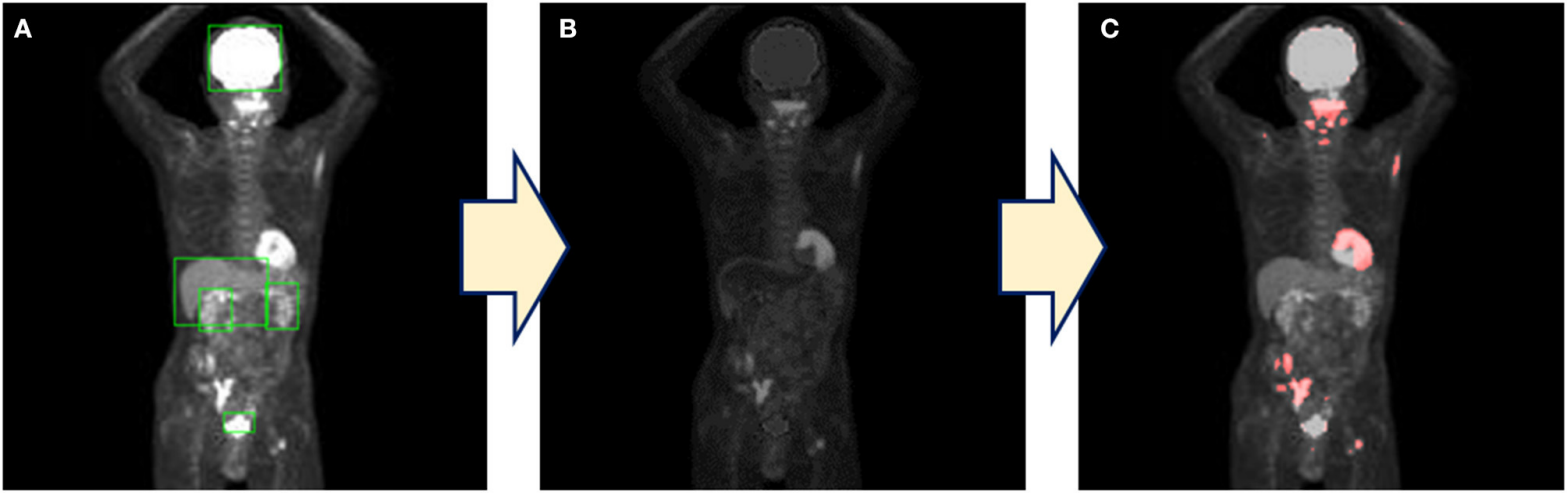
The process of the combination method. **(A)** The detection of physiological uptake by YOLOv2. **(B)** Only physiological uptakes detected by YOLOv2 were subtracted from the original images. **(C)** A color map is generated by coloring the pixels above the threshold.

Each physiological uptake (brain, heart, liver, kidney, and bladder) detected by the created model described above in section **Automatic Detection** (Figure 3A) was cropped from the original MIP image with a five-pixel margin on the coordinate information of the bounding boxes. For the clipped physiological uptake images, we performed threshold processing to set <50% of the maximum pixel values to zero, and we applied a two-dimensional (2D) Gaussian smoothing kernel with a standard deviation of 1.2. The images generated by this process were subtracted from the original MIP images (Figure 3B); the histogram of the images was drawn, and the mode of frequency was determined. The threshold was defined as the value of the pixel value plus 100. Higher uptakes other than physiological uptakes were emphasized in the image by displaying red pixels above the threshold value (Figure 3C). Our new combination method was thus defined as the generation of these images as a color map derived from the series of methods described above.

#### Evaluation of the Created Color Maps

We evaluated the combination method by comparing the abnormal imaging findings between the abnormal findings obtained by two experienced radiologists (SF, 5 years; KH, 18 years) and the color maps generated by the combination method. The radiologist's findings were evaluated according to the presence/absence of abnormalities in each of the seven regions (brain, head/neck, chest, abdomen, pelvis, heart, intestine). When the region colored on the color map corresponded to the region diagnosed as abnormal by the radiologist, it was considered to be correctly detected.

We defined the coverage rate as the percentage of correctly detected abnormalities relative to the radiologist's findings of the presence of abnormalities. A false-positive result was defined as when a site with no abnormalities on the radiologist's findings was colored on the color map. And also, a false-negative result was defined as when the site noted as abnormal by the radiologist was not colored on the color map. We defined the false-positive rate as the ratio of false-positive results to the radiologist's findings of no abnormalities and the false-negative rate as the ratio of false-negative results to the site of colored on the color map. In addition, we obtained false-positive and false-negative rates for each site. These values were calculated for the evaluation of the detection precision of the combination method.

## Results

### Average Precision of Each Class

**Figure 4:**
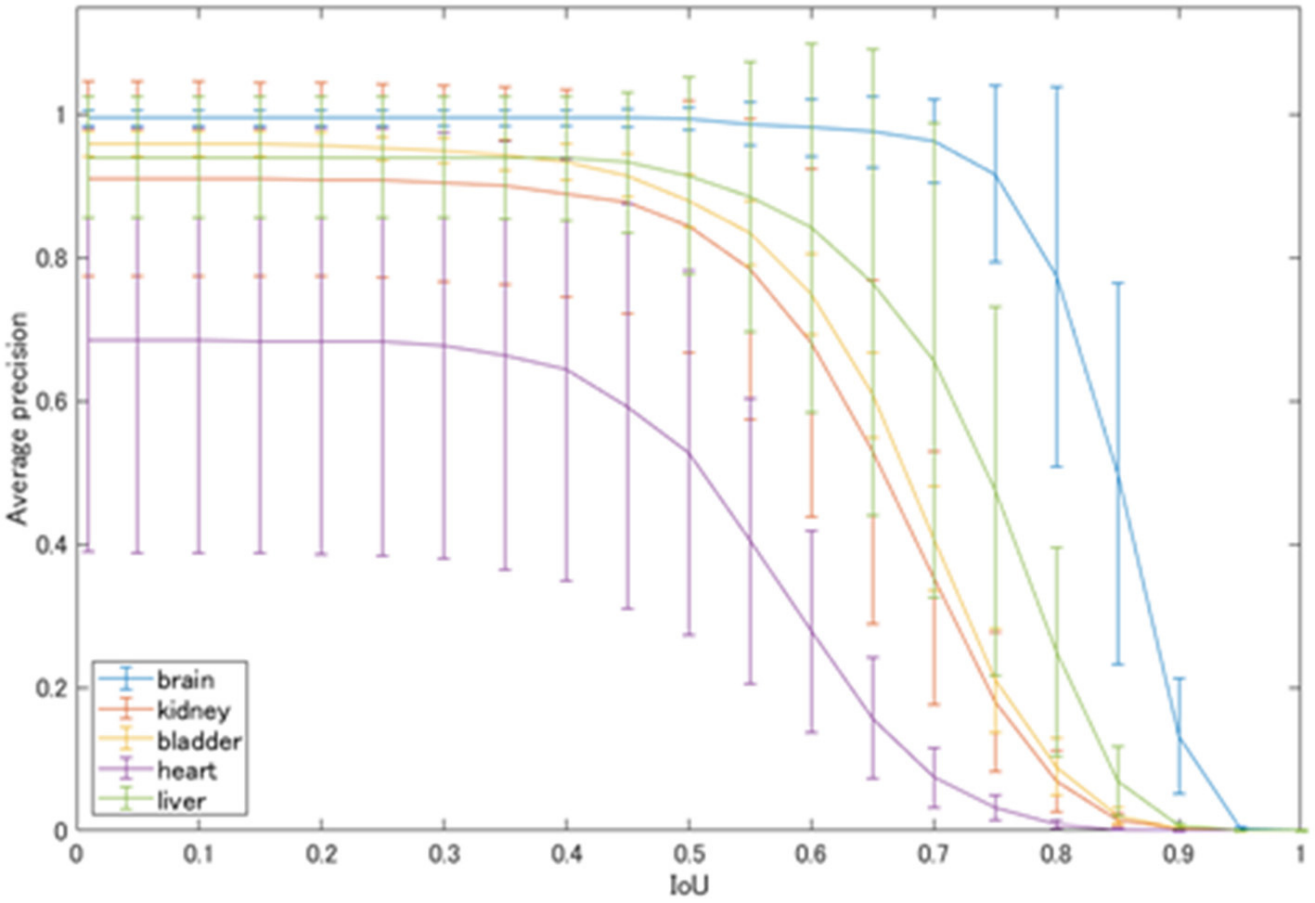
Average precision of each physiological uptake.

The average precision of each class automatically detected by YOLOv2 is shown in Figure 4. At the IoU of 0.5, physiological uptakes in the brain were detected with rather high precision (AP: 0.993), followed by high APs in the liver (0.913), bladder (0.879), and kidneys (0.843). The detection of the cardiac uptakes were the worst, with an AP of 0.527 at the IoU of 0.5.

**Figure 5:**
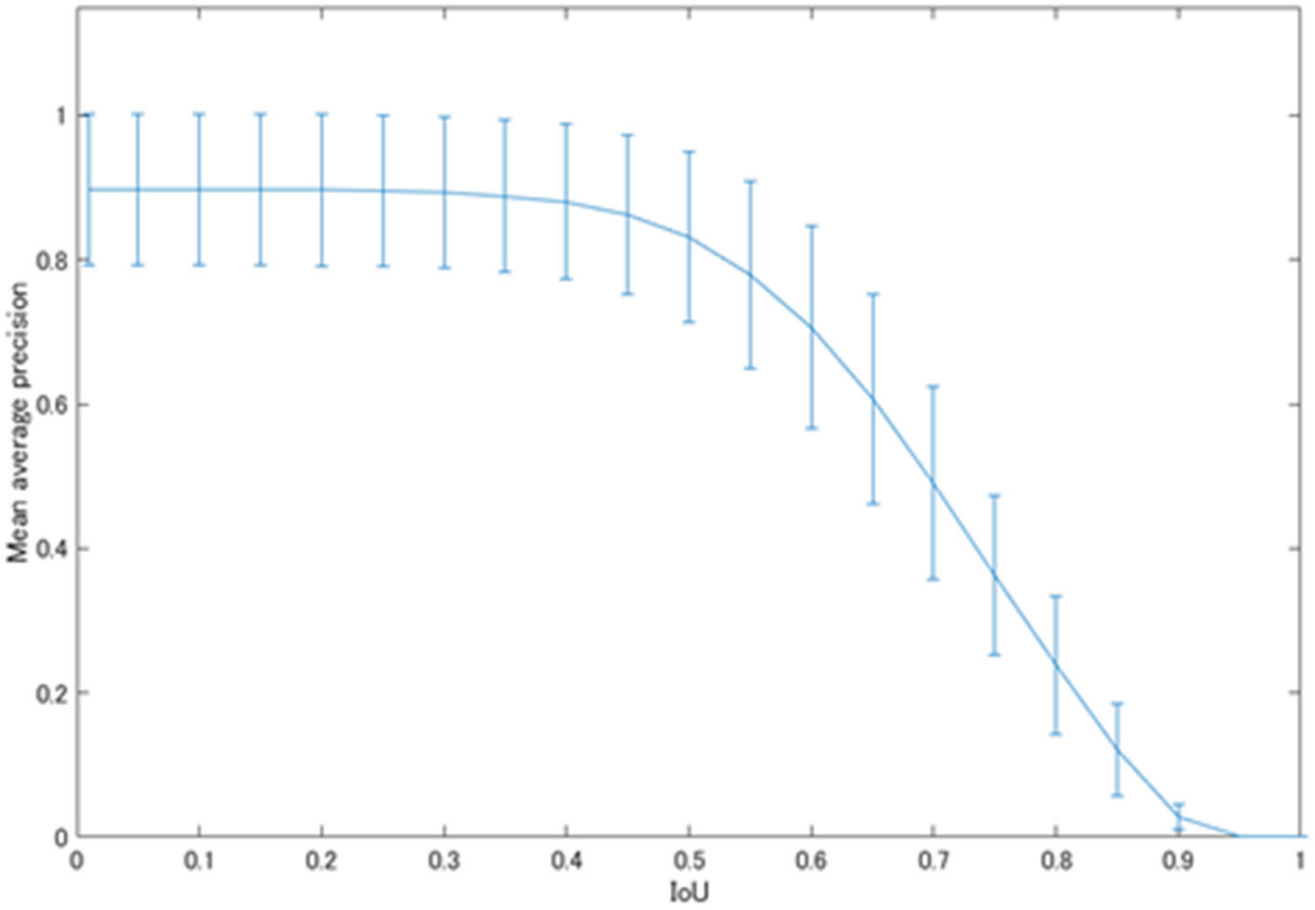
The mAP values of the physiological uptakes.

Figure 5 shows the mean average precision of each physiological uptake (brain, heart, liver, kidney, and bladder) detected by YOLOv2. The mAP value was decreased over the threshold IoU of 0.5. The mAP was 0.831 with the threshold IoU of 0.5. The average FPS for each subset was 31.60 ± 4.66.

### Coverage Rate, False Positive Rate, and False Negative Rate of the Combination Method

Table 1 shows the coverage and false-positive rate of the color maps generated by the combination method. The coverage rate, false-positive rate, and false-negative rate for detecting abnormal uptake were 0.9205 ± 0.0312, 0.3704 ± 0.0213, and 0.1000 ± 0.0774, respectively.

**Table 1:** Coverage rate, false-positive rate, and false-negative rate of the new combination method.

	**Coverage rate**	**False positive rate**	**False negative rate**
Subset A	0.8719	0.3796	0.2310
Subset B	0.9343	0.3356	0.0543
Subset C	0.9554	0.3684	0.0329
Subset D	0.9129	0.3857	0.0970
Subset E	0.9279	0.3828	0.0851
Mean ± SD	0.9205 ± 0.0312	0.3704 ± 0.0213	0.1000 ± 0.0774

Table 2 shows the false-positive rate and false-negative rate by site by the combination method. The false-positive rate was highest in the head/neck, at 0.7629 ± 0.0385. The false-negative rates were higher in the abdomen and head/neck, 0.2047 ± 0.1254 and 0.2000 ± 0.4472, respectively.

**Table 2:** False-positive rate and false-negative rate by site.

	**False positive rate**	**False negative rate**
Brain	0.0137 ± 0.0254	0.2000 ± 0.4472
Head/neck	0.7629 ± 0.0385	0
Chest	0.3523 ± 0.1460	0.0331 ± 0.0197
Abdomen	0.3326 ± 0.1873	0.2047 ± 0.1254
Pelvis	0.1674 ± 0.0341	0.0667 ± 0.0726
Heart	0.0331 ± 0.0296	0.0761 ± 0.0288
Intestine	0.3754 ± 0.0602	0.1198 ± 0.1539

## Discussion

We evaluated the precision of a network model of deep learning for object detection (i.e., YOLOv2) for detecting physiological uptakes on FDG-PET images with a rectangular region, and we developed a combination method using YOLOv2 and subtraction processing for the detection of abnormal uptakes. The detector was created by training with a dataset of 3,500 MIP images with data augmentation processes (such as rotation and zooming) and the mAP of 0.831 with the IoU of 0.5. The average FPS was >30. The results demonstrated high detection precision and a high speed for the detection of physiological uptakes. In particular, the physiological uptakes of the brain could be detected with fairly high precision, with an AP of 0.993 with the IoU of 0.5. The next-highest values were found for the liver, bladder and kidneys at 0.913, 0.879, and 0.843, respectively.

However, as shown in Figure 4, the detection of physiological uptakes in the heart resulted in lower APs compared to those in the other classes. One possible reason for the very high APs (>0.9) in physiological uptakes in the brain and liver compared to the slightly lower APs in other classes is that FDG-PET provides metabolic images rather than anatomical images like CT or magnetic resonance imaging (MRI). The brain and liver showed no differences in the MIP images due to the minimal differences in metabolism between individuals. However, the shape and degree of uptakes of the bladder and kidneys varied greatly on the images, as there were large individual differences in uptakes depending on the degree of urination. In addition, normal heart uptakes were more difficult to diagnose than the other four classes because of the variety of uptake patterns (22), which may have contributed to the low AP for the heart.

With regard to limitations of the detection of physiological uptakes, the number of features for the detector that was necessary for the training of the created models was limited. In other words, for the further improvement of the detection precision, we have to consider increasing the number of training images and the number of patterns of data augmentation because the present study was performed with rotation and zooming of the images. However, our findings demonstrated that higher AP and mAP values could be obtained by varying the degree of rotation and the zoom rate as data augmentation. In addition, regarding the pixel data, the raw data of the DICOM files (which had the dynamic range of pixel values) would be taken into account for the evaluation of the new method's precision because the present study was performed using JPEG images. Moreover, it has been reported that the mAP was improved by color operations and geometric operations (23), and the precision of the mAP could be changed by performing procedures other than those used in the present study.

Regarding the CNN models, although our results showed sufficient precision and response speed for the real-time detection, further improvements of the detection precision and speed may be obtained by using a network model for object detection other than YOLOv2, such as DetectNet (24), Single Shot MultiBox Detector (25), and Faster R-CNN (26). New network models such as Feature Pyramid Networks (27) and Mask R-CNN (28), which are based on Faster R-CNN with additional technologies, have been reported to improve processing speed and average precision. There is also an improved version of YOLOv2 with a deeper network model, YOLOv3 (29). However, the detection precision shown as the mAP was 0.831 with the IoU of 0.5, and the average FPS was over 30 FPS in the present study. These results demonstrated that the detection was sufficiently accurate and faster compared to other studies aimed at real-time detection (30). Therefore, although there is room for further improvement in the detection performance due to factors such as the number of training images, data augmentation, and different network models, we observed that the detection performance obtained herein (including the speed response) was sufficient for use in image diagnoses.

We took advantage of the detection of physiological uptake by the detector created using YOLOv2, an object detection model based on deep learning technology. The combination method was our newly developed method for detecting abnormal uptakes by combining YOLOv2 and a subtraction process. The color maps generated by the combination method showed great merit, with a very high coverage rate of >92% for the abnormal findings identified by the highly experienced radiologist. We used MIP data in this study because of the smaller size of the data and the ability to evaluate the whole body in a single image. The use of MIP images not only reduced the learning time even when the number of images was increased but also provides more information in one image than can be obtained when using tomographic images. For example, because it is often difficult to distinguish between normal and abnormal uptakes in a single tomographic image, the diagnosis is usually made by examining both the upper and lower slices. For these reasons, there are advantages to using MIP images for diagnoses using deep learning.

However, the false-positive rate obtained by this detector was ~37%, which is not very low; this result might be due to the use of MIP images with 2D data. The generation of a false high uptake might be caused by overlapping of low uptake when the three-dimensional (3D) structure of the body was rendered into a 2D image, and this effect might be the reason for the increase of false-positive results. It was thus difficult to reduce the number of false positives with MIP images, and there was a limit to the precision of the combination method with MIP data. Incorporating tomographic data could be one solution. Moreover, the false-positive rate by site was the highest in the head/neck. As for the neck region, Purohit BS reported (31) that FDG uptake by normal lymphoid tissue act as a confounding factor for the diagnosis of neck tumors. This was the reason we did not define the uptakes at this site as a physiological uptake. As a result, the false-positive rate was higher than any other site due to detecting physiological uptakes incorrectly. However, the head/neck was the only site with a false-negative rate of zero and did not miss the lesion. Regarding to the false-negative rate, it was higher in the abdomen and brain because abnormal uptakes within physiological uptakes were more common in these sites than in other sites, and these uptakes could not be detected.

Although we applied the dataset to the 2D network model as YOLOv2 due to the limitation of computer resources, there are 3D network models (32–36) that can be used with one-time training with the whole data. We have also considered these 3D network models for the evaluation of precision in a future study. Furthermore, we have to take into consideration changing window width of the MIP images to distinguish between physiological uptakes and tumors because the MIP technique has a limitation to detect abnormal uptakes within physiological uptakes. However, in light of the accurate detection of the physiological uptakes observed herein and the coverage rate for abnormal uptake indicated by a radiologist, our present results have established this combination method as a useful diagnostic tool with real-time detection.

## Conclusions

We investigated the precision of the detection of physiological uptakes and developed a combination method for diagnoses based on FDG-PET images. With the use of using YOLOv2, the physiological uptake of FDG on MIP images was automatically detected with high precision and high speed. In addition, the combination method, which utilizes the characteristics of the detector by YOLOv2, detected abnormalities identified by the experienced radiologist with a high coverage rate. The combination method's detection performance and fast response demonstrated its usefulness as a diagnostic aid tool.

## Data Availability Statement

The original contributions generated for this study are included in the article/supplementary materials, further inquiries can be directed to the corresponding author/s.

## Ethics Statement

The studies involving human participants were reviewed and approved by Hokkaido University Hospital Ethics Committee. Written informed consent from the participants' legal guardian/next of kin was not required to participate in this study in accordance with the national legislation and the institutional requirements.

## Author Contributions

MK contributed to data analysis and the writing and editing of the article. KH, SF, and KK proposed the idea and reviewed and edited the paper. HS contributed to algorithm construction, performed supervision, and reviewing and editing. KM contributed to data acquisition and reviewed and edited the paper. CK performed supervision and project administration. All authors have read and agreed to the published version of the manuscript.

## Conflict of Interest

The authors declare that the research was conducted in the absence of any commercial or financial relationships that could be construed as a potential conflict of interest.
